# The Crosstalk between Osteoclasts and Osteoblasts Is Dependent upon the Composition and Structure of Biphasic Calcium Phosphates

**DOI:** 10.1371/journal.pone.0132903

**Published:** 2015-07-20

**Authors:** Yukari Shiwaku, Lynn Neff, Kenichi Nagano, Ken-Ichi Takeyama, Joost de Bruijn, Michel Dard, Francesca Gori, Roland Baron

**Affiliations:** 1 Department of Oral Medicine, Infection and Immunity, Harvard School of Dental Medicine, Boston, MA, United States of America; 2 Department of Medicine, Endocrine Unit, Massachusetts General Hospital, Harvard Medical School, Boston, MA, United States of America; 3 Xpand Biotechnology BV, Bilthoven, The Netherlands; 4 Department of Periodontology and Implant Dentistry, New York University College of Dentistry, New York, NY, United States of America; Université de Lyon - Université Jean Monnet, FRANCE

## Abstract

Biphasic calcium phosphates (BCPs), consisting of hydroxyapatite (HA) and β-tricalcium phosphate (β-TCP), exhibit good biocompatibility and osteoconductivity, maintaining a balance between resorption of the biomaterial and formation of new bone. We tested whether the chemical composition and/or the microstructure of BCPs affect osteoclasts (OCs) differentiation and/or their ability to crosstalk with osteoblasts (OBs). To this aim, OCs were cultured on BCPs with HA content of 5, 20 or 60% and their differentiation and activity were assessed. We found that OC differentiation is partially impaired by increased HA content, but not by the presence of micropores within BCP scaffolds, as indicated by TRAP staining and gene profile expression. We then investigated whether the biomaterial-induced changes in OC differentiation also affect their ability to crosstalk with OBs and regulate OB function. We found that BCPs with low percentage of HA favored the expression of positive coupling factors, including sphingosine-kinase 1 (SPHK1) and collagen triple helix repeat containing 1 (Cthrc1). In turn, the increase of these secreted coupling factors promotes OB differentiation and function. All together our studies suggest that the chemical composition of biomaterials affects not only the differentiation and activity of OCs but also their potential to locally regulate bone formation.

## Introduction

Synthetic biomaterials are routinely used as bone substitutes in orthopedic surgery to repair large bone defects caused by tumors or trauma [1,2] and in oral surgery for alveolar ridge augmentation and treatment of infrabony periodontal defects [3]. Autografts however still remain the gold standard for bone repair, substitution and augmentation followed by allografts, but both have major drawbacks that include limited availability, morbidity associated with the donor site, and potential transmission of pathogens in the case of allografts [4]. Therefore there is still a strong need to develop synthetic materials to avoid these limitations.

The rationale behind the use of calcium phosphate (CaP) materials as bone substitutes is that their composition is similar to that of the mineral phase of bone, including some key properties of bone, such as biodegradability, bioactivity and osteoconductivity [1,5]. CaP materials are classified based on their composition as: hydroxyapatite (HA), Ca_10_(PO_4_)_6_(OH)_2_; beta-tricalcium phosphate (β -TCP), Ca_3_(PO_4_)_2_ and biphasic calcium phosphate (BCP), an intimate mixture of HA and β-TCP of varying HA/β-TCP weight ratio. The composition of the CaP material has direct consequences on its performance, including its ability to be resorbed by osteoclasts (OCs) [6]. When osteoclasts precursors are plated on CaP materials and their differentiation induced, they generate multinucleated TRAP positive osteoclasts that form an acting ring and resorb the materials, creating resorption pits [7]. However, resorption of HA is usually limited, with few and only superficial pits in contrast with BCP with low HA/TCP ratio and β-TCP that exhibit more frequent, deeper and better delimited pits [6,8,9]. Furthermore, OC differentiation and activity are affected by the other physicochemical properties of CaPs, such as surface roughness, surface topography and crystallinity. [10–14].

The function of OCs is not however limited to their ability to resorb bone. In the context of bone remodeling OCs also contribute to bone formation through their ability to communicate with osteoblasts (OBs) in a crosstalk that regulates the local recruitment and bone forming activity of OBs, in a process called coupling [15,16]. During coupling, osteoblast precursors migrate to the resorption lacunae created by OCs and start forming new bone, constituting the basis of the bone remodeling sequence [17]. In this process, OCs locally control OB recruitment and differentiation through the secretion of coupling factors [15,18] that can either promote or inhibit OB differentiation. We and others have shown that Sphingosine-1-phosphate (S1P), produced by sphingosine kinase (SPHK) in OCs, promotes OB differentiation and mineralization [19–21]. Other cytokines (clastokines) secreted by mature OCs, such as BMP-6, Wnt10b, collagen triple helix repeat containing 1 (Cthrc1) and complement component 3a (C3a) have also been reported to enhance OB differentiation [20,22,23]. Furthermore, EphrinB2 (EfnB2) ligands on OCs may be coupled with EphB4 receptors on OBs, resulting in accelerated OB differentiation [24]. In contrast, Semaphorin4D (Sema4D), another clastokine produced by OCs, inhibits OB differentiation [25]. Taken together, these findings indicate that coupling factors released by OCs play an important role in the local recruitment, differentiation and activity of OBs. Although it has been proposed that coupling can occur when CaPs are implanted in bony defects [26], whether this process occurs when OCs are attached to biomaterials and how the composition of the materials affects the coupling activity of OCs is not known.

In this study, we hypothesized that a crosstalk between OCs and OBs occurs while these cells are attached on biomaterials such that the process of bone regeneration mimics bone remodeling. To test this hypothesis we investigated whether chemical composition and micropore structure of different CaPs influence OC differentiation and coupling function and thereby OB differentiation. Our results indicate that the ability of OCs to regulate OB differentiation through the secretion of coupling factors is differentially affected by the chemical composition of biomaterials, with decreasing HA content favoring coupling. On the other hand, the presence of micropores does not appear to affect the OC-OB coupling function, even though it may promote sealing zone formation.

## Materials and Methods

### Synthesis of BCP disks

Four different types of BCPs (5HA porous, 5HA dense, 20HA dense, and 60HA dense) (Table 1) were prepared using two distinct methods. 5HA porous and 5HA dense were prepared with synthesis method A and made by wet precipitation of apatite powder (Ca/P molar ratio = 1.5) with diluted H_2_O_2_ solution then sintered at 1050°C or 1100°C to impart porous and dense surface features, respectively. The cylinders were processed into disks (9 x 1 mm) using a lathe and a diamond-coated saw microtome (Leica SP1600, Leica Microsystems, IL, USA). Cylinders cleaned in successive ultrasonic baths of acetone, ethanol, and deionized water.

**Table 1:** Chemical composition and porosity of BCP disks.

	Chemical composition (HA/β-TCP)	Micropores	Disk diameter (mm)	Disk thickness (mm)	Synthesis Method
5HA porous	5/95	+	9	1	A
5HA dense	5/95	-	9	1	A
20HA dense	20/80	-	15	1	B
60HA dense	60/40	-	15	1	B

20HA dense and 60HA dense were manufactured from BCP powders using synthesis method B. The powders were synthesized by reaction of calcium hydroxide and phosphoric acid in an aqueous solution. To obtain the different Ca/P ratio’s for BCP, different ratios of calcium hydroxide and phosphoric acid were used. The suspension was spray dried using a VSD6.3R Spraydryer with rotary atomizer (GEA Westfalia Separator Group, Oelde, Germany). This powder was pressed at 4000 MPa in order to form dense blocks of BCP. Blocks were sintered at 1100°C for over 6 h and then cut into uniformly sized (15 mm diameter, 1 mm height) pieces using a diamond hollow drill.

### Characterization of BCP disks

Chemical compositions and crystal strictures of the BCP disks were determined from X-ray diffraction (XRD). XRD was analyzed using Miniflex (Rigaku, Tokyo, Japan). XRD patterns of BCP disks were recorded by step-scanning at 0.02° intervals from 25° to 41° with Cu X-ray tube at 30 kV and 15 mA and Ni-filter. Surface topography of the BCPs was characterized using the Field Emission Scanning Electron Microscope (FE-SEM; Zeiss Supra55VP Field Emission Scanning Electron Microscope, Oberkochen, Germany) after sputter coating them with gold for grain and pore size measurement.

### Mice

Mice were housed at the Harvard Medical School and all animal experimental protocols were approved by the Harvard Institutional Animal Care and Use Committee (IACUC). Mice were allowed free access to water and a maintenance diet and maintained on a 12 hours light/12 hours dark cycle. 13 male and 11 pregnant C57Bl6 mice (Charles River Laboratories, MA, USA) were used in osteoclast and osteoblast culture. Animals euthanized by CO_2_ inhalation followed by cervical dislocation and bone harvest. For osteoclast culture, cells were isolated from 1 mouse and replated on materials in each experiment. The supernatants of osteoclast cultures were pooled and applied for conditioned media experiments. For osteoblast culture, littermates were used in each experiment.

### Primary osteoclast culture

Primary mouse bone marrow macrophages (BMM) were obtained by flushing femurs and tibias of 6- to 8-week-old male C57Bl/6 mice. Cells were cultured in α-minimum essential medium (αMEM, Life Technologies, Grand Island, NY, USA) with 10% fetal bovine serum (FBS), 1% GlutaMAX, 100 U/ml penicillin, 100 μg/ml streptomycin (all obtained from Life Technologies) and 30 ng/ml M-CSF (R&D, Minneapolis, MN, USA) at 37°C in humidified 5% CO_2_. After 3 days, BMMs were replated on the different BCP disks at a density of 1.0 x 10^5^ cells/ml. All materials were sterilized by ultraviolet irradiation for 1 h (30 min for each side of disks) and preincubated for 1 h in α-MEM with 10% FBS before seeding cells. Cells were cultured for an additional 7 days with 30 ng/ml M-CSF and 10 ng/ml RANKL (R&D) to induce osteoclast differentiation. Medium was changed every other day and supernatants collected as conditioned media at day 4 after RANKL stimulation. Conditioned media were centrifuged to remove the cell debris and stored at -80°C.

### Tartrate-resistant acid phosphatase (TRAP) staining

OCs cultured on BCPs were fixed with 3.7% formaldehyde in phosphate-buffered saline (PBS) for 15 min. After washing with PBS, cells were stained with Fast Red Violet LB (Sigma, St. Louis, MO,USA) dissolved in TRAP solution for 15 min at 37°C. TRAP solution consisted of 100 mM sodium acetate, 50 mM sodium tartrate, Naphthol AS-MX phosphate, and NN dimethyl formamide (all obtained from Sigma) and adjusted pH to 5.0. TRAP positive multinucleated cells (with 2 or more nuclei) on BCPs were observed using a microscope (Leica MZFLIII Microscope).

### Assessment of actin organization in osteoclasts by confocal microscopy

For immunofluorescence labeling, cells were fixed with 3.7% formaldehyde in PBS for 10 min. BCP disks were rinsed twice with PBS and blocked with 1% bovine serum albumin (BSA, Sigma), 0.05% Triton (Sigma), and 5% normal goat serum (Gemini Bio-Products, West Sacramento, CA, USA) in PBS for 30 min. Samples were incubated with anti-Integrin beta-3 antibody (1:100 dilution, Millipore) in blocking solution for 2 h. After washing with PBS, samples were incubated with rhodamine phalloidin (1:50 dilution, Life Technologies) and Alexa Fluor 488 goat anti-rabbit IgG secondary antibody (1:1000 dilution, Life Technologies) for 1 h. Nuclei were stained using DAPI (Cell signaling). Cells were imaged with a Zeiss LSM 510 two-photon laser-scanning microscope. Quantification of podosome rings and filopodia was performed using Image J.

### Primary osteoblast culture, differentiation, and mineralization

Primary mouse calvarial cells were isolated from 3-day-old C57Bl/6 mice. Briefly, calvariae were dissected and sequentially digested for 90 min in a collagenase solution containing type I and II collagenase (Worthington, Lakewood, NJ, USA) at 37°C. The supernatants of the first and second digestion were discarded and those after third digestion were collected. Calvarial cells were cultured in α-MEM with 10% FBS, 100 U/ml penicillin, 100 μg/ml streptomycin at 37°C in humidified 5% CO_2_.

For OB differentiation assays, calvarial cells were replated at 1.2 x 10^5^ or 2.0 x 10^4^ cells/ml in 48 well plates and differentiation was initiated 24 hours after plating by replacing the growth medium with a media consisting of 50% of conditioned media collected from OC cultures on BCPs and 50% of α-MEM with 10% FBS, and antibiotics, including 50 μg/ml ascorbic acid (Sigma) and 5 mM beta-glycerophosphate (Merck Millipore, Darmstadt, Germany).

Alkaline phosphatase (ALP) activity was measured using FAST-p-Nitrophenyl Tablets (Sigma). Briefly, cells were lysed in 10 mM Tris pH 7.4, 0.2% IGEPAL, and 100 mM phenylmethylsulfonyl fluoride (all obtained from Sigma). After sonication and centrifugation, ALP activity was determined in the supernatants and normalized to protein content analyzed by Pierce BCA protein assay kit (Thermo Scientific, Waltham, MA, USA). For bone nodule formation visualization, cells were fixed with 70% ethanol at -20°C and stained with 40 mM Alizarin red stain solution (Sigma, pH = 4.2).

### RNA isolation and quantitative real-time PCR

RNA was isolated from osteoclasts on BCPs using the TRIzol Reagent (Life Technologies) following the manufacturer’s protocol. For osteoblasts cultured with osteoclastic conditioned media, RNA was isolated using RNeasy Plus Mini kit (Qiagen, Hilden, Germany). Reverse transcription was performed using SuperScript III First-Strand Synthesis System for RT-PCR (Life Technologies). Quantitative real-time PCR expression analysis was performed using a FastStart Universal SYBR Green Master (Rox) (Roche), StepOnePlus Real-time PCR System (Life Technologies) and iCycler iQ Multicolor Real-Time Detection System (Bio-Rad). Beta-2-microglobulin (B2M) was used for normalization. The primer sets used in PCR are shown in Table 2.

**Table 2:** Primer sequences used in quantitative real-time PCR analysis.

Gene	Forward	Reverse
NFATc1	AGGCTGGTCTTCCGAGTTCA	ACCGCTGGGAACACTCGAT
CTSK	CAGCTTCCCCAAGATGTGAT	AAAAATGCCCTGTTGTGTCC
TRAP	TCCTGGCTCAAAAAGCAGTT	ACATAGCCCACACCGTTCTC
EfnB2	TCTGTGTCATCGGTTGGCTACGTT	ACAGACGCACAGGACACTTCTCAA
SPHK1	TGAGGTGGTGAATGGGCTAATGGA	AACAGCAGTGTGCAGTTGATGAGC
SPHK2	TGGGCTGTCCTTCAACCTCATACA	AGTGACAATGCCTTCCCACTCACT
Cthrc1	CAGTTGTCCGCACCGATCA	GGTCCTTGTAGACACATTCCATT
Sema4D	CCTGGTGGTAGTGTTGAGAAC	GCAAGGCCGAGTAGTTAAAGAT
Runx2	TGCCCAGGCGTATTTCAG	TGCCTGGCTCTTCTTCTGAG
OCN	TCTGACAAACCTTCATGTCC	AAATAGTGATACCGTAGATGCG
B2M	CTGCTACGTAACACAGTTCCACCC	CATGATGCTTGATCACATGTCTCG

### Statistical analysis

All experiments were repeated more than three times and Figures showed the representative data of single culture. Results are expressed as the mean ± standard deviation (SD) and p values less than 0.05 were considered statistically significant. One-way analysis of variance (ANOVA) was used for each experiment to compare the means among the groups. If the ANOVA was significant, then the Tukey HSD test was used as a post-hoc test.

## Results

### Characterization of BCP disks

**Fig 1:**
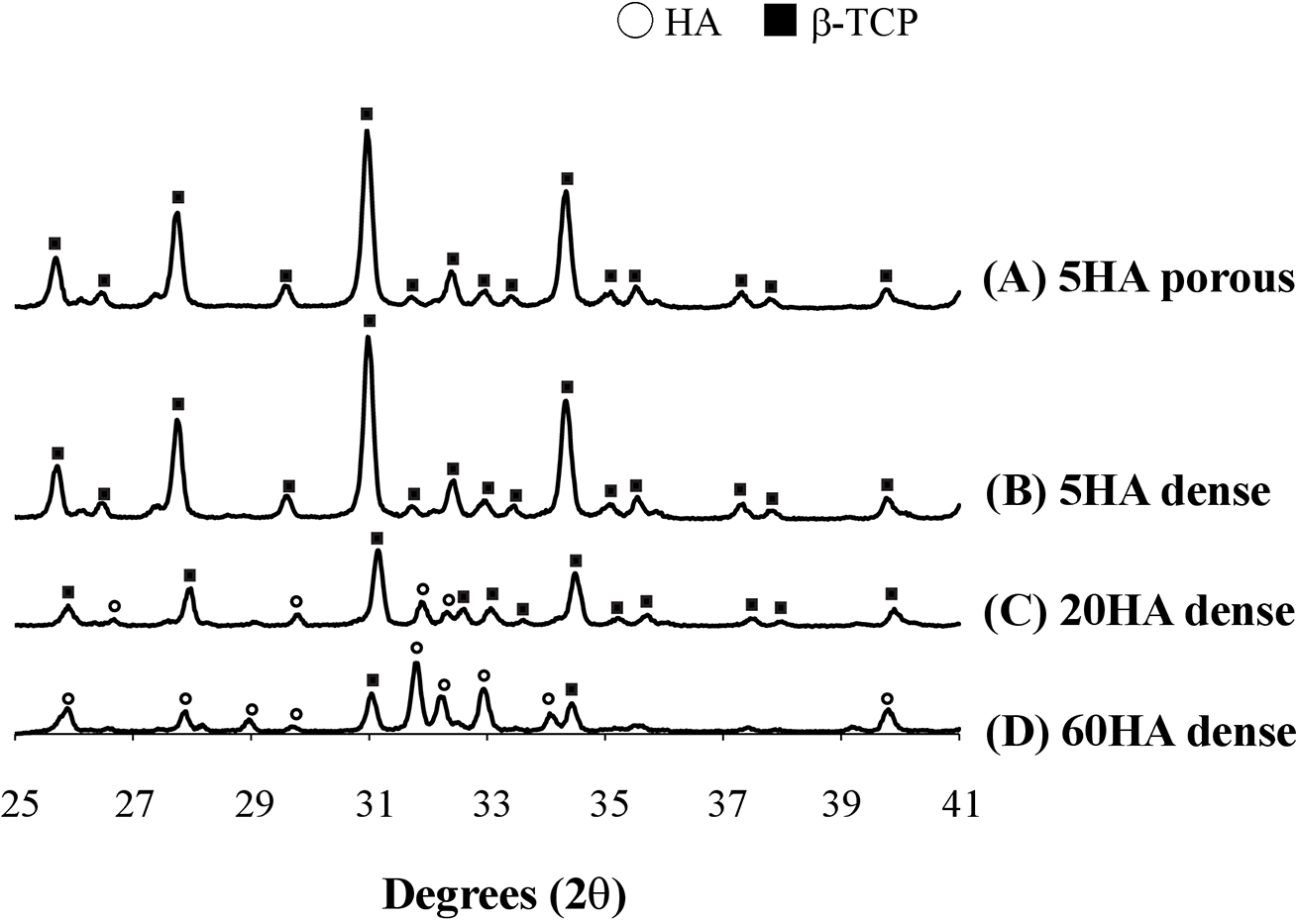
XRD patterns of BCP disks. (A) 5HA porous, (B) 5HA dense, (C) 20HA dense and (D) 60HA dense.

The chemical composition of BCPs was characterized by XRD (Fig 1). XRD patterns of 5HA dense and 5HA porous showed highly crystallized phases and possessed the typical reflection of β-TCP [27,28]. 60HA dense and 20HA dense displayed the peaks of both β-TCP and HA. Compared to 20HA dense, 60HA dense possesses more apatitic peaks.

**Fig 2:**
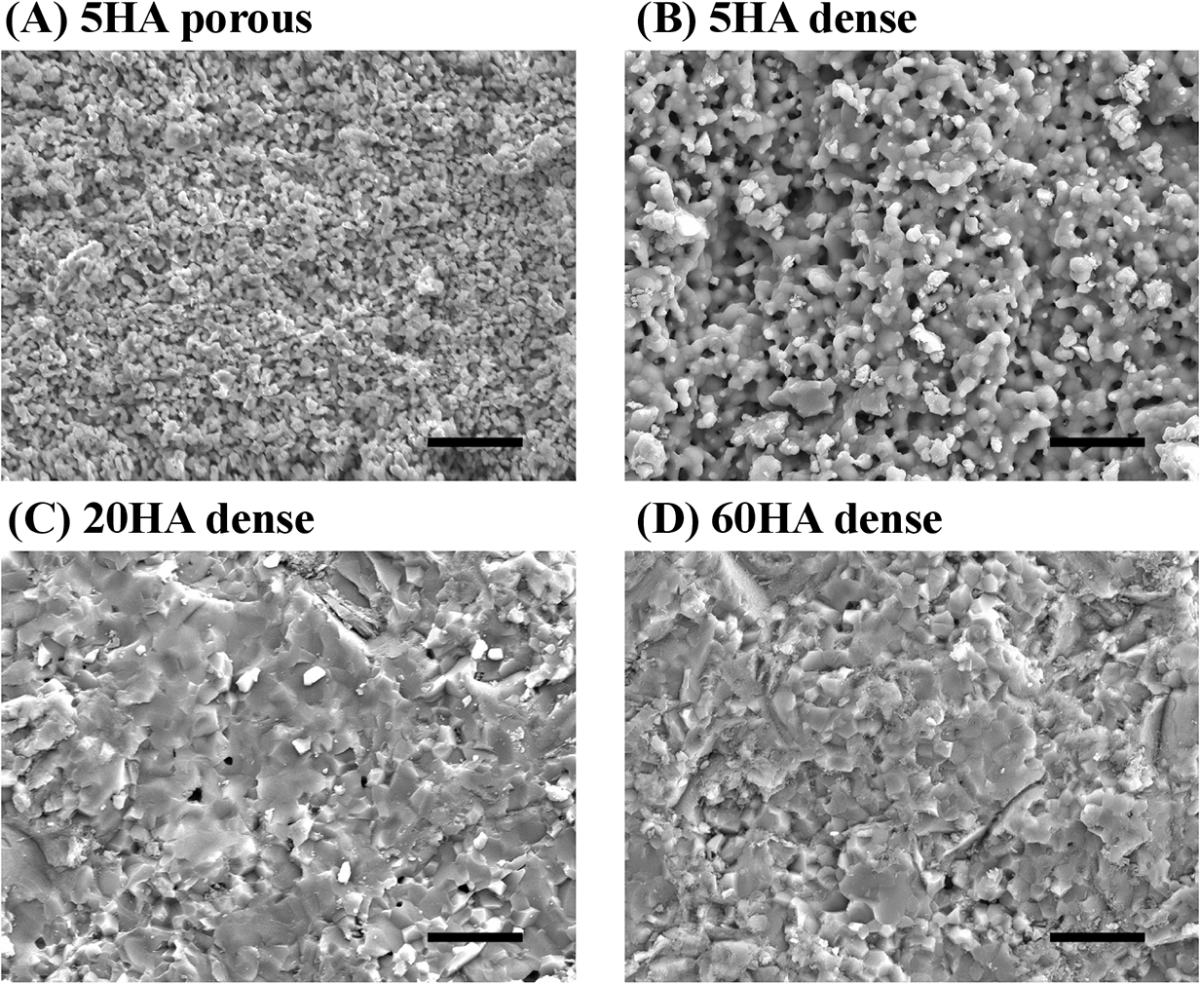
FE-SEM photographs of BCP disks. (A) 5HA porous, (B) 5HA dense, (C) 20HA dense and (D) 60HA dense. Bars = 10 μm.

We next examined the surface topography of each BCP using FE-SEM (Fig 2). 5HA dense and 5HA porous have small and fine crystals, 2~10 μm or 0.5~2 μm in length, respectively. In contrast, 60HA dense and 20HA dense have larger crystals, forming dense contacts between crystals and crystal boundary zone. There were few pores between grains in the surfaces of 60HA dense and 20HA dense. 5HA dense showed more large pores between grains but, as expected, 5HA porous has many more but much smaller micropores, with a diameter of approximately 1~4 μm. The difference in porosity and grain size also affected the surface roughness of these materials. 5HA porous had smoother topography (Ra < 0.13 μm) than 5HA dense (Ra > 1 μm).

### Osteoclast differentiation on BCP disks with different chemical composition and structure

**Fig 3:**
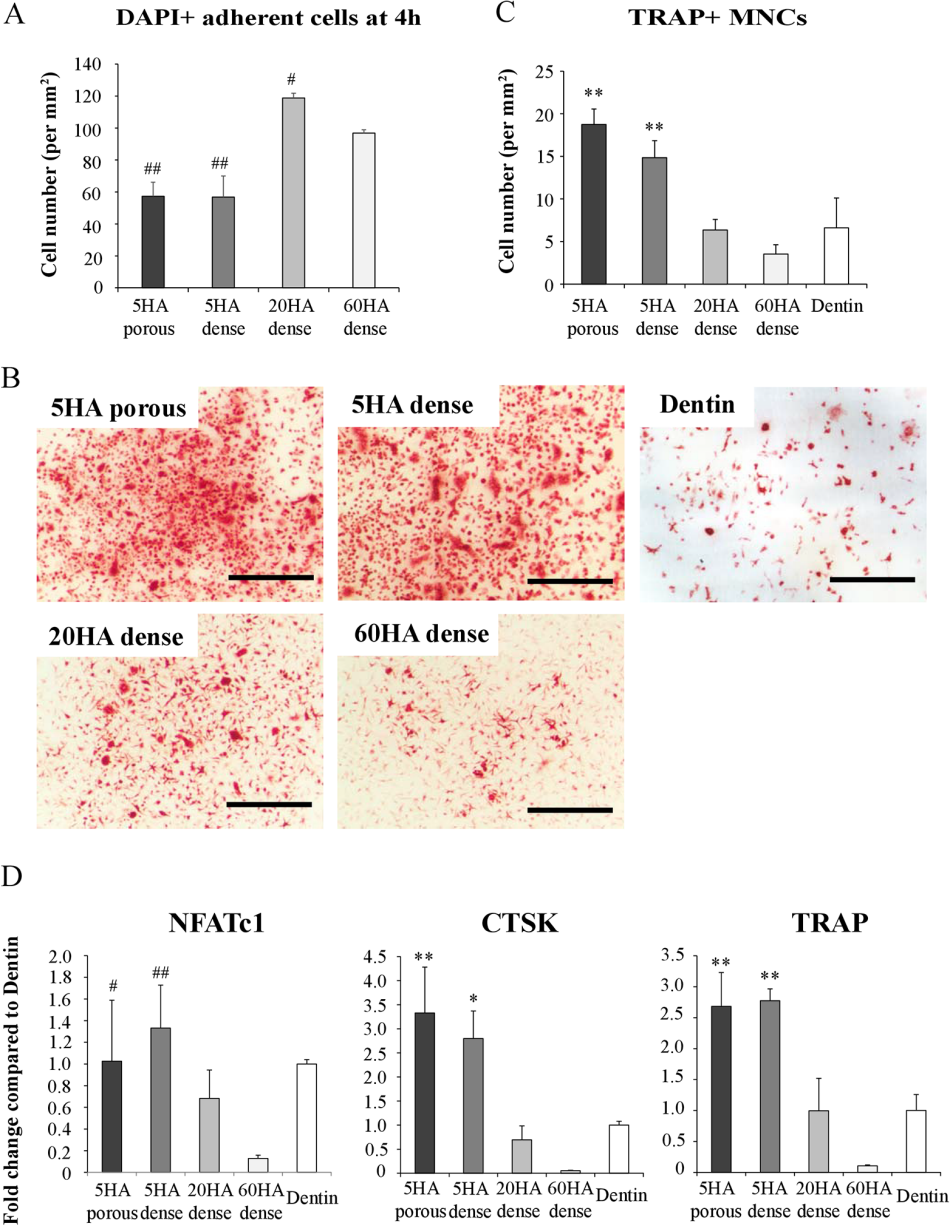
Osteoclast differentiation on different BCP disks and dentin. (A) The number of DAPI positive cells attached on BCPs at 4 hour after seeding BMMs. Data are expressed as the mean ± S.D. n = 3. ^##^p<0.01 and ^#^p<0.05 significant difference from 60HA dense. (B) TRAP staining of osteoclasts on BCPs at day7 after replating BMMs in the presence of M-CSF and RANKL. Bar = 500 μm. (C) The number of TRAP positive cells with 2 or more nuclei was counted 7 days after plating BMMs on each BCP disk and dentin. Data are expressed as the mean ± S.D. n = 3. **p<0.01 significant difference from dentin (control). (D) Expression of osteoclast markers (NFATc1, CTSK, and TRAP) at day7 on BCPs and dentin. Data are expressed as the mean ± S.D. n = 3. **p<0.01 and *p<0.05 significant difference from dentin (control). ^##^p<0.01 and ^#^p<0.05 significant difference from 60HA dense.

To evaluate the influence of BCPs chemical composition and micropores on OC differentiation, bone marrow macrophages (BMMs) were plated on the different biomaterials and differentiated in the presence of M-CSF and RANKL. Plating of cells on plastic or on dentin slices was used as controls. Although the number of cells plated in each condition was the same, more cells attached to the 20HA and the 60HA than on the 5HA disks after 4 hours (Fig 3A). Despite the fact that less cells initially adhered to the 5HA disks, we detected significantly more OCs (TRAP-positive multinucleated cells, TRAP^+^ MNCs) on both 5HA dense and 5HA porous (Fig 3B and 3C) at the end of the culture than on 20HA and on 60HA dense, the latter showing the lowest number of TRAP^+^ MNCs on its surface.

To confirm these findings, we expanded the analysis to the expression of OC markers in these cells, such as nuclear factor of activated T-cells cytoplasmic 1 (NFATc1), TRAP and cathepsin K (CTSK), using quantitative real-time PCR. We used dentin slices as a control that mimics a physiologic mineralized substrate. As shown in Fig 3D, the expression of CTSK and TRAP was significantly higher in OCs cultured on 5HA dense and 5HA porous substrates compared to those cultured on dentin. In contrast, cells cultured on 60HA dense failed to show increased expression of OC specific genes. Taken together, these findings suggest that the higher the percentage of HA in BCPs, the lower the ability of OC precursors to differentiate into mature OCs. No significant differences were observed between 5HA dense and 5HA porous suggesting that the presence of micropores or difference in roughness within BCPs did not influence OC differentiation.

### Cytoskeletal organization of osteoclasts on BCPs

To evaluate the behavior of osteoclasts on the different BCPs we examined the cytoskeletal organization of cells plated on dentin or on the different biomaterials tested. When osteoclast precursors differentiate into mature OCs, they form clusters of punctate actin-rich adhesion structures enriched in integrin receptors called podosomes, which then form small rings, later organized in a podosome belt/sealing zone at the cell’s periphery [29,30].

**Fig 4:**
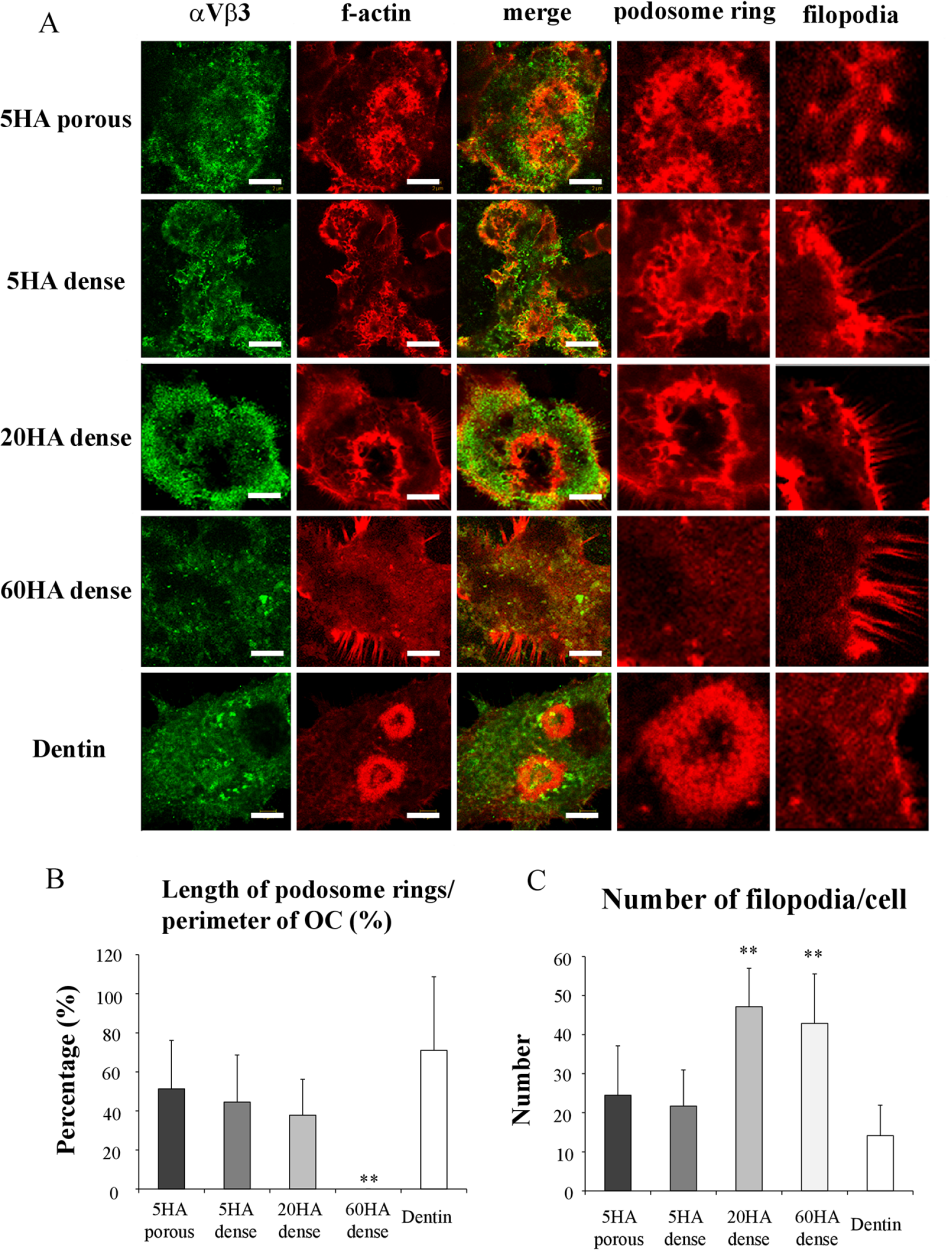
Formation of podosome rings in osteoclasts cultured on BCPs. (A) Cells were labeled for nuclei (blue), f-actin (red) and αVβ3 integrin (green). Bars = 10 μm. Right two columns are pictures of podosome rings or filopodia in high magnification. (B) Percentage of covered with podosome rings in perimeter of osteoclasts cultured on BCPs. Data are expressed as the mean ± S.D. n = 6–8. **p<0.01 significant difference from dentin (control). (C) Number of filopodia of osteoclasts cultured on BCPs. Data are expressed as the mean ± S.D. n = 6–8. **p<0.01 significant difference from dentin (control).

Immunofluorescent labeling of f-actin and αVβ3 integrin show well-organized podosome rings (Fig 4A), in OCs cultured on dentin. Comparable structures were formed on both 5HA BCPs and on 20HA dense BCP. In contrast, podosome rings and belts were absent from cells plated on 60HA dense BCP (Fig 4B). Higher magnification of the cell’s periphery demonstrated that podosome-like structures were absent from cells plated on dentin (all podosomes were concentrated in the rings and belts that formed) whereas some podosomes appeared at the periphery of cells plated on 5HA BCPs. Importantly, podosomes were totally absent from cells plated on 60HA BCP and there was instead a progressive increase in the number of filopodia as the concentration of HA present in the BCP increased (Fig 4B and 4C). These findings indicate that the characteristic functional cytoskeletal organization of OCs with formation of podosome belts is impaired when cells are cultured on BCPs that contain a higher concentration of HA compared to OCs cultured on 5HA dense and 5HA porous BCPs.

### Effects of BCPs on the expression of coupling factors by osteoclasts

**Fig 5:**
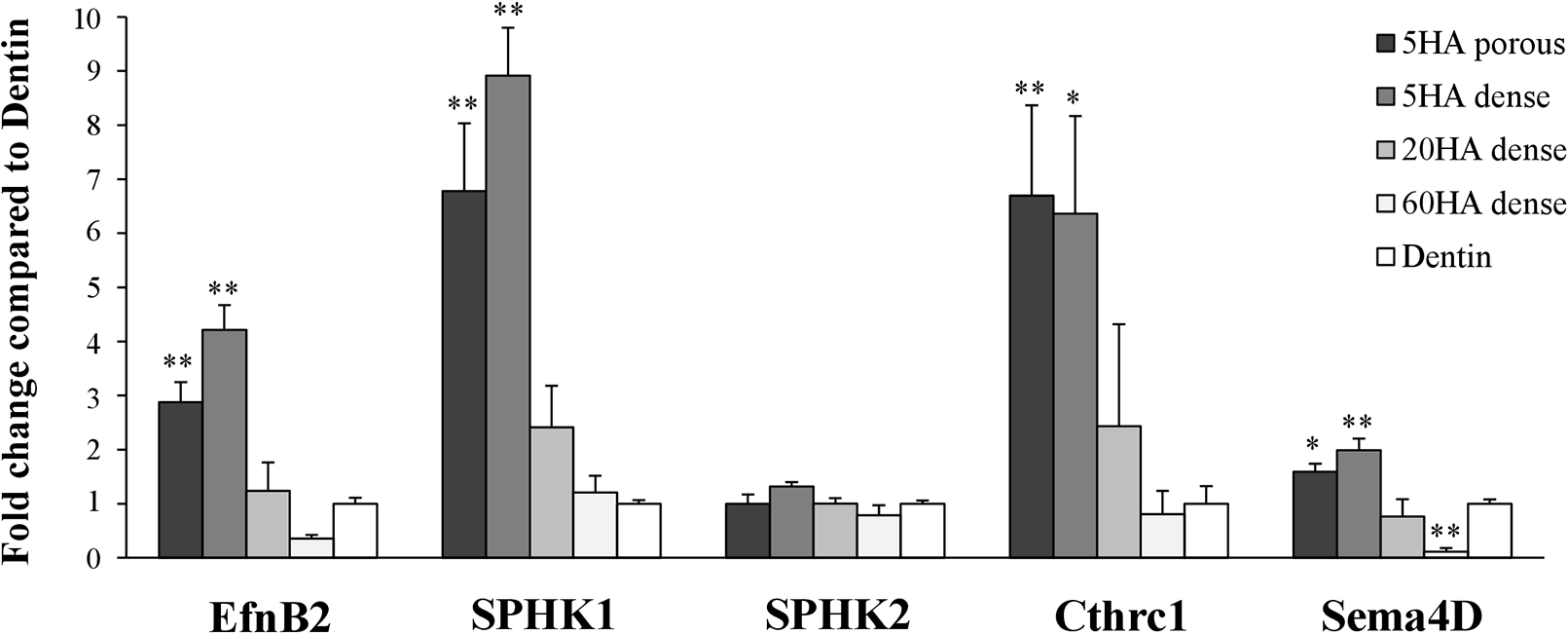
Expression of coupling factors (EfnB2, Cthrc1, SPHK1/2 and Sema4D) in osteoclasts cultured on BCPs and dentin at day7. Data are expressed as the mean ± S.D. n = 3. **p<0.01 and *p<0.05 significant difference from dentin (control).

Given that our data indicated that both OC differentiation and cytoskeletal organization are affected by the HA/β-TCP ratio, we then asked whether the different BCPs also regulate the crosstalk between OCs and OBs, possibly by affecting the ability of individual OCs to express and/or secrete coupling factors. To address this question, we first examined the expression of coupling factors known to regulate OB differentiation either positively or negatively, normalized to housekeeping genes and therefore reflecting expression/cell independent of the number of cells present on the substrate (Fig 5). Among the positive coupling factors investigated, the expression of EfnB2 and SPHK1 was significantly higher in OCs cultured on 5HA than in those cultured on 20HA or 60HA (p<0.01). The expression of Cthrc1 in OCs cultured on 5HA was also significantly higher than in OCs cultured on 60HA (p<0.05). Strikingly, the expression of Cthrc1 was 6 times higher and that of SPHK1 was 9 times higher in OCs plated on 5HA dense compared to OCs plated on dentin. These results suggest that the expression of coupling factors by OCs is affected by the composition of BCPs. In contrast to SPHK1, SPHK2 expression was not affected by the composition of BCPs, suggesting a gene-specific response. On the other hand, we detected only very low levels of expression of BMP6 and Wnt10b in OCs on all BCPs (data not shown). Of note, the expression of the negative coupling factor Sema4D was also higher in OCs cultured on 5HA than on 20HA and was lower than the levels observed on dentin in OCs cultured on 60HA. However, the expression of Sema4D in OCs cultured on 5HA dense increased only 2 fold compared to dentin, suggesting that the expression of positive coupling factors such as SPHK1 or Cthrc1 are dominant over that of negative coupling factor like Sema4D.

Since the level of expression of all these coupling factors, with the exception of SPHK2, was higher in the OCs cultured on 5HA than when cultured on dentin, these results suggest that this type of BCP actually induces the expression of coupling factors above and beyond a more natural substrate such as dentin matrix.

### Effects of BCPs on the secretion of OC coupling factors to affect osteoblasts

**Fig 6:**
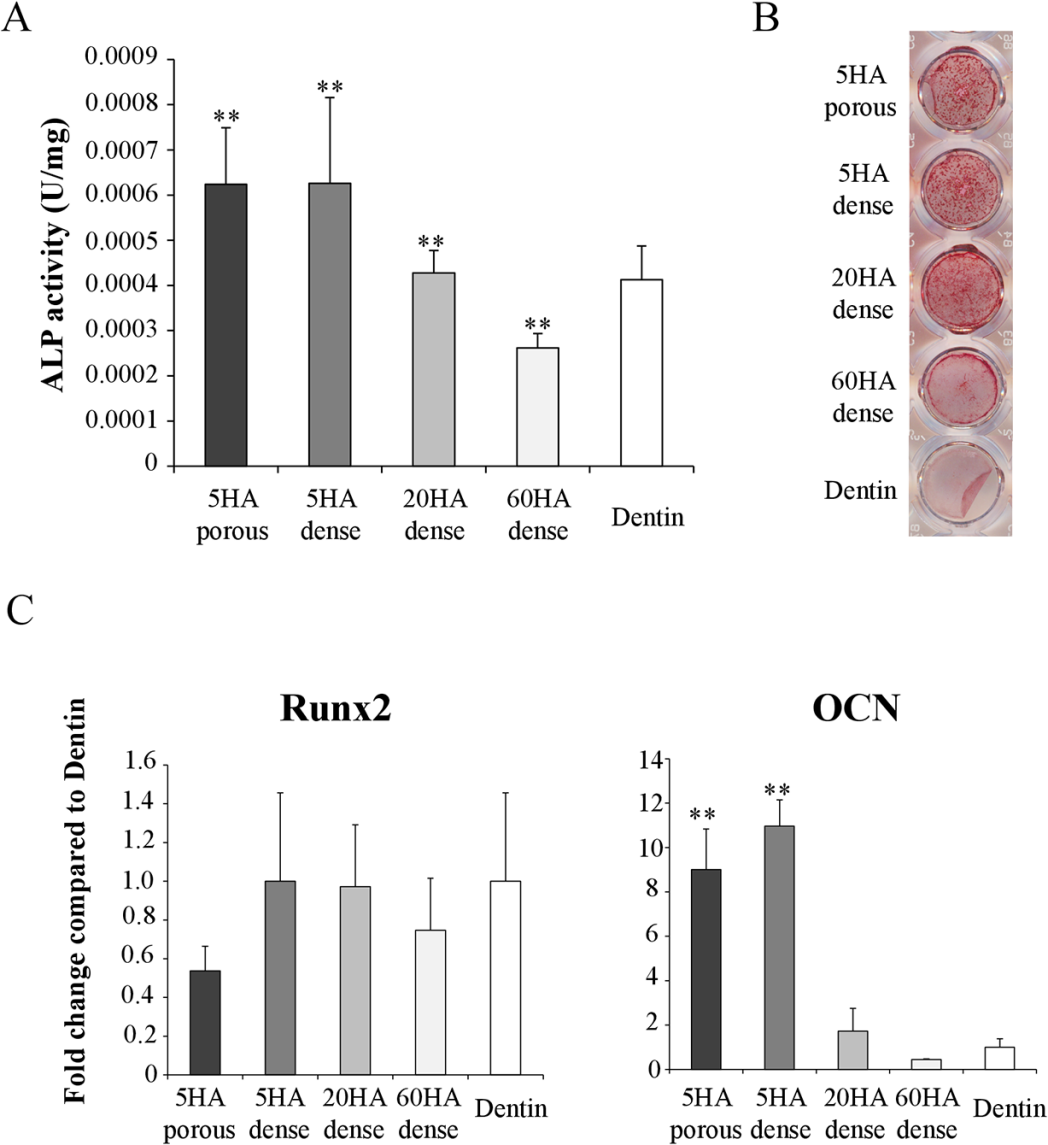
The effect of osteoclastic conditioned media collected from BCPs on osteoblast differentiation. (A) ALP activity at day7. Data are expressed as the mean ± S.D. n = 3. **p<0.01 significant difference from dentin (control). (B) Alizarin red staining at day14. (C) Relative gene expression of osteoblast markers (Runx2 and OCN) at day7. The expression levels have been normalized with housekeeping gene B2M. Data are expressed as the mean ± S.D. n = 3. **p<0.01 and *p<0.05 significant difference from dentin (control).

Based on our findings that OCs cultured on biodegradable BCPs (5HA dense and porous) express higher levels of both positive (EfnB2, SPHK1 and Cthrc1) and negative coupling factor (Sema4D) compared to OCs cultured on 60HA dense and 20HA dense, we then asked whether these factors were indeed secreted and active by testing whether conditioned media (CM) harvested from OCs cultured on the different BCPs, and thereby expressing different levels of coupling factors, affect differentially OBs. To this end, we cultured primary calvarial OB in media containing 50% of CM collected from the OC cultured on the various BCPs (Fig 6).

When OBs were treated with media harvested from wells containing the different materials but without OCs no alizarin red staining was observed (data not shown). In contrast, CM from BCP disks on which OCs had been plated showed significant alterations of OB differentiation markers and mineralization in vitro. In agreement with our observations on the expression of the coupling factors, OC conditioned media collected from cells cultured on 5HA dense and 5HA porous increased ALP activity significantly compared with dentin and promoted more Alizarin Red positive bone nodule formation relative to that collected from cells cultured on 60HA (Fig 6A and 6B). In parallel experiments the expression of the early OB marker Runx2 was not affected by CMs of OCs cultured on various materials compared to dentin. In contrast, Osteocalcin (OCN), a marker of mature OBs, was higher in OBs cultured with conditioned media from OCs cultured on 5HA dense and 5HA porous than on 20HA and 60HA (p<0.01) (Fig 6C).

Taken together, these results demonstrate that OCs cultured on different BCPs secrete a variable amount and mix of coupling factors in their CM, differentially affecting OB differentiation and mineralization. Our findings suggest that the effect of positive coupling factors is dominant in CM from OCs cultured on 5 HA BCPs, resulting in enhanced OB differentiation and mineralization. Two important observations are that 1) The higher the HA contained in the BCP, the lower the induction of OB differentiation and nodule formation; and 2) OCs cultured on BCPs with only 5% HA induce significantly more coupling factor-dependent induction of OB differentiation and nodule formation than OCs cultured on dentin.

## Discussion

Our study demonstrates that OCs behave differently on different biomaterials and this affects not only their differentiation and cytoskeletal organization, but also their ability to communicate with cells of the OB lineage and to induce bone formation, at least in vitro. These findings have important conceptual and practical consequences. First, they place the link between resorbability and osteoconductivity in the biological context of the remodeling sequence, where OCs and matrix resorption not only precede but also induce local bone formation on their substrate. Second, they illustrate the fact that the chemical composition of a substrate influences the ability of OCs to enhance OB differentiation and bone formation, i.e. the coupling activity of OCs. Third, they demonstrate that not all substrates are equal in their ability to enhance OC support of OBs: BCPs containing lower percentage of HA are better inducers of OB-enhancing coupling factors than BCPs with high percentages of HA.

The goal of these studies was to study whether physicochemical properties (chemical composition and structure) of CaPs affects OC differentiation and/or activity and more importantly to assess the effects of biomaterials on the OC-OB crosstalk. In agreement with several previous studies [6,9,31], we found that the increased presence of HA in the substrate decreases OC differentiation and impairs cytoskeletal organization, while others have shown that it also impairs resorption.

In terms of differentiation of the osteoclasts, both 5HA dense and 5HA porous, although they initially had a lower number of adherent cells, allowed a better differentiation of BMMs into osteoclasts than 20HA and 60HA dense, despite the fact that the cells were plated under identical conditions (Fig 3A and 3B). Interestingly, it was more that 5HA, independent of its microstructure, enhanced OC differentiation above a natural substrate like dentin, whereas 20HA and 60HA behaved in a manner comparable to dentin (Fig 3C). However, in addition to affecting negatively the number of OCs formed in these assays, increasing the concentration of HA to 60% as in 60HA negatively affected the differentiation process of OC precursors. Indeed, NFATc1, TRAP and CTSK expression was lower in 60HA compared to dentin, whereas 20HA behaved similarly to dentin and 5HA enhanced the expression of these three markers significantly. Thus, the lower the HA content in these BCPs, the higher the number of OCs that are formed and the faster they differentiate. Among physical parameters, the surface roughness (Ra) of each BCP could also play a role in OC differentiation or activity. 5HA porous had smoother surface (Ra = 0.126 μm) than 5HA dense (Ra = 1.287 μm), but despite this 10X difference in roughness 5HA dense and 5HA porous behaved in very similar ways, suggesting that roughness was not a significant player in our system. Surface roughness of 20HA dense and 60HA dense was similar to that of 5HA dense, and therefore cannot explain the observed differences. Thus, although surface topography has been reported to affect osteoclast activity it seems that the difference of surface roughness between the four BCPs did not affect osteoclast differentiation or coupling properties.

A marker of the functional organization of OCs is the formation of the sealing zone when plated on a resorbable substrate. The sealing zone is a specialized area of the membrane and the cell cortex of mature and active OCs and consists of a dense array of interconnected podosomes [32], specialized actin-rich attachment complexes used by OCs, dendritic cells, macrophages, and other cells from the monocytic lineage to adhere to their substrate and migrate on its surface [33]. The formation of podosomes and their organization into a sealing zone are critical for efficient bone resorption, as exemplified by the reduced or absent bone resorption by OCs when podosome components are deleted or sealing zone formation is compromised [34]. The molecular signals that induce the formation of the sealing zone are not fully understood, but are likely mediated by integrins and more specifically by the vitronectin receptor heterodimer αVβ3 [35]. The mineral content of bone itself could trigger OCs polarization and sealing zone formation [36]. Our results suggest that the chemical composition of BCPs also influences the cytoskeletal organization of OCs, their ability to form podosomes and the formation of the sealing zone. As clearly shown on Fig 4, podosomes and podosome rings (which precede the formation of a true sealing zone) are formed on dentin, 20HA dense and 5HA BCPs. However, no podosomes are observed on 60HA dense. Conversely, the higher the HA content the more filopodia are formed by the OCs, such that on 60HA the cells form no podosomes but only stress fibers. Interestingly, the microstructure of the material seemed to also affect podosome and ring formation, with the 5HA porous providing the closest cytoskeletal organization to dentin (Fig 4). It therefore appears that high HA content leads to a perturbation of the cytoskeletal organization of OCs with an inability to form podosomes and sealing zones, a change expected to impair resorption, as suggested by the literature [6,9].

It is however not possible to determine if the reduced podosome sealing zone formation on BCPs containing higher HA is the consequence of overall diminished OC differentiation or the result of defective signaling originating from receptors that sense the composition or crystalline organization of the mineral since the nanotopography of a material can affect the adhesion of OCs through integrins, altering interactions with their ligands and receptor clustering [37], with some topographies favoring sealing zone formation [38]. As mentioned, it is only based on the criteria of podosome formation that we found the porous 5HA material to possibly be more favorable than the 5HA dense material. In addition, the microstructure of TCP and HA affects resorption by OCs [39,40]. To fully distinguish the influence of the three-dimensional texture from that of the surface chemistry would require a more precise analysis of the micro and nano topography of the CaP materials tested here.

The second and most important aim of our study was however to test whether the different materials would affect the ability of OCs to support OB differentiation and activity and mimic the in vivo coupling process observed during the remodeling of bone. Although the existence of a coupling process and crosstalk between OCs and OBs has been shown many years ago [17], it is only recently that the mechanisms by which this crosstalk occurs have been explored [15,18], but never in relation to biomaterials and this despite the observation that resorption of a CaP material and the deposition of bone around it are coupled [41]. BCPs containing low HA promote bone regeneration in vivo [42] and induce the formation of multinuclear OC-like cells around the new bone area [43]. Furthermore, bisphosphonates, which target the OCs, also reduce the bone formation induced by osteoinductive ceramics [44,45], further suggesting the importance of coupling in bone regeneration using biomaterials.

Several coupling factors synthetized and secreted by OCs and that can act on OBs have been identified [15,18]. Our first approach was to determine how the different BCP materials tested in this study affected the expression of selected coupling factors in OCs. Among the coupling factors known to positively promote OB differentiation, we found that the expression of EfnB2, Cthrc1, and SPHK1 was increased significantly in OCs cultured on BCP with low HA compared with dentin, with the 20HA showing intermediate levels of expression. Of particular interest are our findings on SPHK1, an enzyme that acts upon sphingosine to generate S1P [19], an essential secreted coupling factor [20,21,46]. In OBs, S1P interacts with S1P receptors and promotes OB migration and survival [19,20,46,47]. It also promotes OB differentiation by activating Wnt/β-catenin and BMP2 signaling [48,49] and OC differentiation by regulating RANKL expression via Cox-2 and PGE2 [19]. It is therefore plausible that in our study S1P is responsible in part for the observed activation of both OB and OC differentiation, explaining our results with the 5HA biomaterial. Cthrc1 is also a soluble protein expressed by mature bone-resorbing OCs, which has been shown to regulate osteoblast differentiation and thereby bone formation [22].

Interestingly, we also found that the expression of Sema4D, a negative coupling factor shown to inhibit OB differentiation [25], is higher in OCs cultured on BCPs with low HA compared to OCs cultured on high HA percentage. However, since the expression of Sema4D was increased only by 2-fold in OCs cultured on low HA BCPs, while the expression of Cthrc1 and SPHK1 increased by 6 and 8 fold, respectively, the function of the positive coupling factors secreted by OCs cultured on degradable BCPs is dominant over the activity of the negative ones, as clearly demonstrated by the overall stimulatory effects of OC conditioned media in OB cultures.

## Conclusion

In conclusion, our results show for the first time that the composition of the biomaterials directly affects not only the ability of OC precursors to differentiate into mature OCs, but also the ability of these mature OCs to express and secrete clastokines that in turn influence OBs and bone formation. Although, the release of bone formation promoting factors by OCs is not the only mechanism that determines the bioactivity of the CaP material and its clinical success, insights into the mechanism by which the chemical composition of the material regulates OC behavior could help develop materials that favor bone formation. Further studies will be necessary to investigate the expression and secretion of coupling factors when CaP biomaterials are implanted in vivo.

## References

[1] Hannink G, Arts JJ. Bioresorbability, porosity and mechanical strength of bone substitutes: what is optimal for bone regeneration? Injury. 2011;42 Suppl 2:S22–25. 10.1016/j.injury.2011.06.008 21714966

[2] Bose S, Tarafder S. Calcium phosphate ceramic systems in growth factor and drug delivery for bone tissue engineering: a review. Acta biomaterialia. 2012;8:1401–1421. 10.1016/j.actbio.2011.11.017 22127225 PMC3418064

[3] Reynolds MA, Aichelmann-Reidy ME, Branch-Mays GL, Gunsolley JC. The efficacy of bone replacement grafts in the treatment of periodontal osseous defects. A systematic review. Annals of periodontology / the American Academy of Periodontology. 2003;8:227–265. 14971256 10.1902/annals.2003.8.1.227

[4] Cancedda R, Giannoni P, Mastrogiacomo M. A tissue engineering approach to bone repair in large animal models and in clinical practice. Biomaterials. 2007;28:4240–4250. 17644173 10.1016/j.biomaterials.2007.06.023

[5] LeGeros RZ. Calcium phosphate-based osteoinductive materials. Chemical reviews. 2008;108:4742–4753. 10.1021/cr800427g 19006399

[6] Yamada S, Heymann D, Bouler JM, Daculsi G. Osteoclastic resorption of calcium phosphate ceramics with different hydroxyapatite/beta-tricalcium phosphate ratios. Biomaterials. 1997;18:1037–1041. 9239465 10.1016/s0142-9612(97)00036-7

[7] Schilling AF, Linhart W, Filke S, Gebauer M, Schinke T, Rueger JM, et al Resorbability of bone substitute biomaterials by human osteoclasts. Biomaterials. 2004;25:3963–3972. 15046886 10.1016/j.biomaterials.2003.10.079

[8] Benahmed MD, Heymann D, Berreur M, Cottrel M, Godard A, Daculsi G, et al Ultrastructural study of degradation of calcium phosphate ceramic by human monocytes and modulation of this activity by HILDA/LIF cytokine. The journal of histochemistry and cytochemistry: official journal of the Histochemistry Society. 1996;44:1131–1140. 8813078 10.1177/44.10.8813078

[9] Monchau F, Lefevre A, Descamps M, Belquin-myrdycz A, Laffargue P, Hildebrand HF. In vitro studies of human and rat osteoclast activity on hydroxyapatite, beta-tricalcium phosphate, calcium carbonate. Biomolecular engineering. 2002;19:143–152. 12202175 10.1016/s1389-0344(02)00023-0

[10] Gomi K, Lowenberg B, Shapiro G, Davies JE. Resorption of sintered synthetic hydroxyapatite by osteoclasts in vitro. Biomaterials. 1993;14:91–96. 8382090 10.1016/0142-9612(93)90216-o

[11] Costa-Rodrigues J, Fernandes A, Lopes MA, Fernandes MH. Hydroxyapatite surface roughness: complex modulation of the osteoclastogenesis of human precursor cells. Acta biomaterialia. 2012;8:1137–1145. 10.1016/j.actbio.2011.11.032 22178652

[12] Kim HM, Kim YS, Woo KM, Park SJ, Rey C, Kim Y, et al Dissolution of poorly crystalline apatite crystals by osteoclasts determined on artificial thin-film apatite. Journal of biomedical materials research. 2001;56:250–256. 11340596 10.1002/1097-4636(200108)56:2<250::aid-jbm1092>3.0.co;2-s

[13] Doi Y, Iwanaga H, Shibutani T, Moriwaki Y, Iwayama Y. Osteoclastic responses to various calcium phosphates in cell cultures. Journal of biomedical materials research. 1999;47:424–433. 10487896 10.1002/(sici)1097-4636(19991205)47:3<424::aid-jbm19>3.0.co;2-0

[14] Davison NL, ten Harkel B, Schoenmaker T, Luo XM, Yuan HP, Everts V, et al Osteoclast resorption of beta-tricalcium phosphate controlled by surface architecture. Biomaterials. 2014;35:7441–7451. 10.1016/j.biomaterials.2014.05.048 24927681

[15] Sims NA, Martin TJ. Coupling the activities of bone formation and resorption: a multitude of signals within the basic multicellular unit. BoneKEy reports. 2014;3:481 10.1038/bonekey.2013.215 24466412 PMC3899560

[16] Henriksen K, Karsdal MA, Martin TJ. Osteoclast-derived coupling factors in bone remodeling. Calcified tissue international. 2014;94:88–97. 10.1007/s00223-013-9741-7 23700149

[17] Frost HM. Tetracycline-based histological analysis of bone remodeling. Calcified tissue research. 1969;3:211–237. 4894738 10.1007/BF02058664

[18] Martin TJ, Sims NA. Osteoclast-derived activity in the coupling of bone formation to resorption. Trends in molecular medicine. 2005;11:76–81. 15694870 10.1016/j.molmed.2004.12.004

[19] Ryu J, Kim HJ, Chang EJ, Huang H, Banno Y, Kim HH. Sphingosine 1-phosphate as a regulator of osteoclast differentiation and osteoclast-osteoblast coupling. The EMBO journal. 2006;25:5840–5851. 17124500 10.1038/sj.emboj.7601430 PMC1698879

[20] Pederson L, Ruan M, Westendorf JJ, Khosla S, Oursler MJ. Regulation of bone formation by osteoclasts involves Wnt/BMP signaling and the chemokine sphingosine-1-phosphate. Proceedings of the National Academy of Sciences of the United States of America. 2008;105:20764–20769. 10.1073/pnas.0805133106 19075223 PMC2603259

[21] Lotinun S, Kiviranta R, Matsubara T, Alzate JA, Neff L, Luth A, et al Osteoclast-specific cathepsin K deletion stimulates S1P-dependent bone formation. The Journal of clinical investigation. 2013;123:666–681. 10.1172/JCI64840 23321671 PMC3561821

[22] Takeshita S, Fumoto T, Matsuoka K, Park KA, Aburatani H, Kato S, et al Osteoclast-secreted CTHRC1 in the coupling of bone resorption to formation. The Journal of clinical investigation. 2013;123:3914–3924. 10.1172/JCI69493 23908115 PMC3754269

[23] Matsuoka K, Park KA, Ito M, Ikeda K, Takeshita S. Osteoclast-derived complement component 3a stimulates osteoblast differentiation. Journal of bone and mineral research: the official journal of the American Society for Bone and Mineral Research. 2014;29:1522–1530. 10.1002/jbmr.2187 24470120

[24] Zhao C, Irie N, Takada Y, Shimoda K, Miyamoto T, Nishiwaki T, et al Bidirectional ephrinB2-EphB4 signaling controls bone homeostasis. Cell metabolism. 2006;4:111–121. 16890539 10.1016/j.cmet.2006.05.012

[25] Negishi-Koga T, Shinohara M, Komatsu N, Bito H, Kodama T, Friedel RH, et al Suppression of bone formation by osteoclastic expression of semaphorin 4D. Nature medicine. 2011;17:1473–1480. 10.1038/nm.2489 22019888

[26] Mastrogiacomo M, Papadimitropoulos A, Cedola A, Peyrin F, Giannoni P, Pearce SG, et al Engineering of bone using bone marrow stromal cells and a silicon-stabilized tricalcium phosphate bioceramic: evidence for a coupling between bone formation and scaffold resorption. Biomaterials. 2007;28:1376–1384. 17134749 10.1016/j.biomaterials.2006.10.001

[27] Nakamura M, Hentunen T, Salonen J, Nagai A, Yamashita K. Characterization of bone mineral-resembling biomaterials for optimizing human osteoclast differentiation and resorption. Journal of biomedical materials research Part A. 2013;101:3141–3151. 10.1002/jbm.a.34621 23554241

[28] Raynaud S, Champion E, Bernache-Assollant D, Thomas P. Calcium phosphate apatites with variable Ca/P atomic ratio I. Synthesis, characterisation and thermal stability of powders. Biomaterials. 2002;23:1065–1072. 11791909 10.1016/s0142-9612(01)00218-6

[29] Jurdic P, Saltel F, Chabadel A, Destaing O. Podosome and sealing zone: specificity of the osteoclast model. European journal of cell biology. 2006;85:195–202. 16546562 10.1016/j.ejcb.2005.09.008

[30] Saltel F, Chabadel A, Bonnelye E, Jurdic P. Actin cytoskeletal organisation in osteoclasts: a model to decipher transmigration and matrix degradation. European journal of cell biology. 2008;87:459–468. 10.1016/j.ejcb.2008.01.001 18294724

[31] Albiges-Rizo C, Destaing O, Fourcade B, Planus E, Block MR. Actin machinery and mechanosensitivity in invadopodia, podosomes and focal adhesions. Journal of cell science. 2009;122:3037–3049. 10.1242/jcs.052704 19692590 PMC2767377

[32] Luxenburg C, Geblinger D, Klein E, Anderson K, Hanein D, Geiger B, et al The architecture of the adhesive apparatus of cultured osteoclasts: from podosome formation to sealing zone assembly. PloS one. 2007;2:e179 17264882 10.1371/journal.pone.0000179 PMC1779809

[33] Albiges-Rizo C, Destaing O, Fourcade B, Planus E, Block MR. Actin machinery and mechanosensitivity in invadopodia, podosomes and focal adhesions. Journal of cell science. 2009;122:3037–3049. 10.1242/jcs.052704 19692590 PMC2767377

[34] Bruzzaniti A, Neff L, Sandoval A, Du L, Horne WC, Baron R. Dynamin reduces Pyk2 Y402 phosphorylation and SRC binding in osteoclasts. Molecular and cellular biology. 2009;29:3644–3656. 10.1128/MCB.00851-08 19380485 PMC2698752

[35] Duong LT, Nakamura I, Lakkakorpi PT, Lipfert L, Bett AJ, Rodan GA. Inhibition of osteoclast function by adenovirus expressing antisense protein-tyrosine kinase 2. The Journal of biological chemistry. 2001;276:7484–7492. 11102447 10.1074/jbc.M008368200

[36] Saltel F, Destaing O, Bard F, Eichert D, Jurdic P. Apatite-mediated actin dynamics in resorbing osteoclasts. Molecular biology of the cell. 2004;15:5231–5241. 15371537 10.1091/mbc.E04-06-0522 PMC532006

[37] Arnold M, Cavalcanti-Adam EA, Glass R, Blummel J, Eck W, Kantlehner M, et al Activation of integrin function by nanopatterned adhesive interfaces. Chemphyschem: a European journal of chemical physics and physical chemistry. 2004;5:383–388. 15067875 10.1002/cphc.200301014

[38] Geblinger D, Addadi L, Geiger B. Nano-topography sensing by osteoclasts. Journal of cell science. 2010;123:1503–1510. 10.1242/jcs.060954 20375065 PMC2858020

[39] Okuda T, Ioku K, Yonezawa I, Minagi H, Kawachi G, Gonda Y, et al The effect of the microstructure of beta-tricalcium phosphate on the metabolism of subsequently formed bone tissue. Biomaterials. 2007;28:2612–2621. 17316789 10.1016/j.biomaterials.2007.01.040

[40] Gonda Y, Ioku K, Shibata Y, Okuda T, Kawachi G, Kamitakahara M, et al Stimulatory effect of hydrothermally synthesized biodegradable hydroxyapatite granules on osteogenesis and direct association with osteoclasts. Biomaterials. 2009;30:4390–4400. 10.1016/j.biomaterials.2009.05.002 19481798

[41] Manolagas SC. Birth and death of bone cells: basic regulatory mechanisms and implications for the pathogenesis and treatment of osteoporosis. Endocrine reviews. 2000;21:115–137. 10782361 10.1210/edrv.21.2.0395

[42] Arinzeh TL, Tran T, Mcalary J, Daculsi G. A comparative study of biphasic calcium phosphate ceramics for human mesenchymal stem-cell-induced bone formation. Biomaterials. 2005;26:3631–3638. 15621253 10.1016/j.biomaterials.2004.09.035

[43] Ghanaati S, Barbeck M, Detsch R, Deisinger U, Hilbig U, Rausch V, et al The chemical composition of synthetic bone substitutes influences tissue reactions in vivo: histological and histomorphometrical analysis of the cellular inflammatory response to hydroxyapatite, beta-tricalcium phosphate and biphasic calcium phosphate ceramics. Biomedical materials. 2012;7. 10.1088/1748-6041/7/1/015005 22287541

[44] Ripamonti U, Klar RM, Renton LF, Ferretti C. Synergistic induction of bone formation by hOP-1, hTGF-beta3 and inhibition by zoledronate in macroporous coral-derived hydroxyapatites. Biomaterials. 2010;31:6400–6410. 10.1016/j.biomaterials.2010.04.037 20493522

[45] Tanaka T, Saito M, Chazono M, Kumagae Y, Kikuchi T, Kitasato S, et al Effects of alendronate on bone formation and osteoclastic resorption after implantation of beta-tricalcium phosphate. Journal of biomedical materials research Part A. 2010;93:469–474. 10.1002/jbm.a.32560 19582838

[46] Keller J, Catala-Lehnen P, Huebner AK, Jeschke A, Heckt T, Lueth A, et al Calcitonin controls bone formation by inhibiting the release of sphingosine 1-phosphate from osteoclasts. Nature communications. 2014;5:5215 10.1038/ncomms6215 25333900 PMC4205484

[47] Quint P, Ruan M, Pederson L, Kassem M, Westendorf JJ, Khosla S, et al Sphingosine 1-phosphate (S1P) receptors 1 and 2 coordinately induce mesenchymal cell migration through S1P activation of complementary kinase pathways. The Journal of biological chemistry. 2013;288:5398–5406. 10.1074/jbc.M112.413583 23300082 PMC3581421

[48] Matsuzaki E, Hiratsuka S, Hamachi T, Takahashi-Yanaga F, Hashimoto Y, Higashi K, et al Sphingosine-1-phosphate promotes the nuclear translocation of beta-catenin and thereby induces osteoprotegerin gene expression in osteoblast-like cell lines. Bone. 2013;55:315–324. 10.1016/j.bone.2013.04.008 23612487

[49] Sato C, Iwasaki T, Kitano S, Tsunemi S, Sano H. Sphingosine 1-phosphate receptor activation enhances BMP-2-induced osteoblast differentiation. Biochemical and biophysical research communications. 2012;423:200–205. 10.1016/j.bbrc.2012.05.130 22659743

